# Dalbavancin dosing in a severely underweight woman with prosthetic valve endocarditis: the critical role of proactive therapeutic drug monitoring

**DOI:** 10.1093/jac/dkaf365

**Published:** 2025-09-26

**Authors:** Ambra Barco, Dario Cattaneo, Jessica Cusato

**Affiliations:** Infectious Diseases Unit, Ospedale Maggiore Della Carità, Novara, Italy; Department of Biomedical Sciences, Humanitas University, Pieve Emanuele, Milan, Italy; Unit of Laboratory Medicine, IRCCS Humanitas Research Hospital, Rozzano, Milan, Italy; Department of Medical Sciences, Laboratory of Clinical Pharmacology and Pharmacogenetics, University of Turin, Turin, Italy

Dalbavancin is a long-acting lipoglycopeptide antibiotic with potent activity against Gram-positive bacteria. Approved for acute bacterial skin and skin structure infections, it is increasingly used off-label in prolonged or chronic infections, such as osteoarticular disease and endocarditis.^1,2^ In the last few years, therapeutic drug monitoring (TDM) has emerged as a promising approach to optimize the scheduling of dalbavancin dosing in these specific populations.^3–7^ Emerging evidence demonstrates that dalbavancin pharmacokinetics may be significantly altered in obese patients, potentially necessitating adjustments in dosing frequency.^8,9^ However, the therapeutic management of severely underweight patients remains largely unexplored.

We report the case of a 65-year-old Chinese female patient who required initiation of dalbavancin therapy in November 2024 for infective endocarditis of a biological aortic valve (replaced April 2024) caused by Corynebacterium striatum. She initially received vancomycin, discontinued due to a cutaneous adverse reaction, and was then switched to daptomycin, which was well tolerated. Due to a high surgical risk, the patient was deemed ineligible for repeat cardiac surgery.

At presentation, she weighed 28 kg (body mass index 13.3 kg/m^2^), and exhibited preserved renal function (serum creatinine 0.82 mg/dL CKD-EPI estimated glomerular filtration rate [eGFR]: 75 mL/min) and liver function (serum albumin: 37 g/L).

Following multidisciplinary consultation, a dosing regimen of dalbavancin 1000 mg administered on day 1 and day 8 was initiated. Subsequent dosing intervals were guided based on TDM results, assessing both minimum (Cmin, within 1 h before the injection) and maximum (Cmax, 30–60 min after the end of infusion) dalbavancin plasma concentrations, as previously described.^3,4^ Cmin and Cmax values were incorporated into log-linear regression models by plotting the logarithm of dalbavancin plasma concentrations against time, to estimate individualized timing of subsequent doses, with the aim of maintaining Cmin levels above 8 mg/L (based on a breakpoint MIC of 0125 mg/L).^3–7,9^

The quantification of dalbavancin plasma concentrations was performed using a previously validated liquid chromatography–tandem mass spectrometry method.^10^ The volume of distribution was estimated as the ratio between the dalbavancin dose and the mean Cmax concentration. Written informed consent for all medical procedures and interventions performed in accordance with clinical practice was obtained from the patient.

The patient received seven dalbavancin administrations over a 217-day period. Renal and hepatic function remained relatively stable throughout the observation period, with eGFR values ranging from 75 to 55 mL/min (serum creatinine ranging from 0.82 to 1.06 mg/dL) and serum albumin levels ranging from 37 to 41 g/L. In contrast, a progressive increase in body weight was documented, from 28 to 38 kg, paralleling a gradual improvement in inflammatory markers (C-reactive protein and white blood cell count) and an overall enhancement of the patient’s clinical condition.

The longitudinal profile of dalbavancin Cmin and Cmax concentrations is depicted in Figure 1. All doses were administered within one day of the scheduled injection date. As shown in the figure, Cmin concentrations consistently exceeded 8 mg/L at each TDM assessment. The mean volume of distribution was 2.7 ± 0.4 L, ranging from 2.2 to 3.1 L. A statistically significant positive correlation was observed between volume of distribution and body weight (correlation coefficient *r* = 0.63). The eighth dalbavancin dose was scheduled 50 days after the previous administration. At the last follow-up visit, the patient reported a subjective sense of wellbeing, with no fever or signs of systemic infection. Laboratory tests confirmed the absence of inflammation, with negative inflammatory markers. Furthermore, a PET scan performed 6 months after initiation of dalbavancin therapy demonstrated stability of the infectious process.

**Figure 1.**
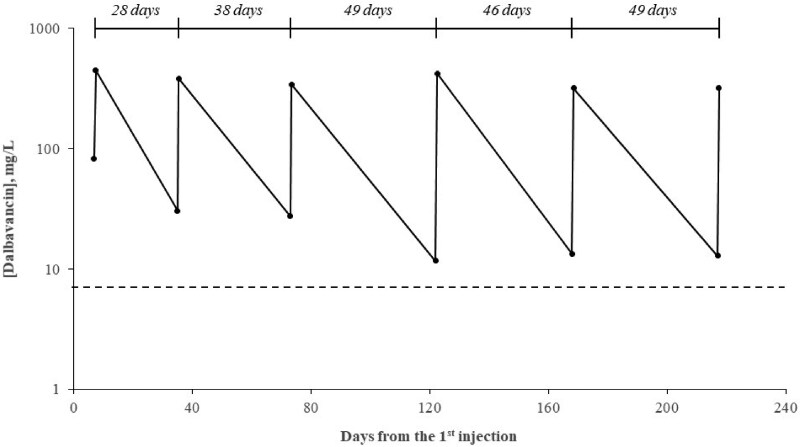
Time-course of dalbavancin minimum and maximum concentrations. The dashed line represents the target concentration (8 mg/L). Dalbavancin concentrations are presented using a semi-logarithmic scale.

Previous studies reported that overweight and obese patients exhibit lower dalbavancin plasma concentrations and increased volumes of distribution compared to individuals with normal body weight.^8,9^ In this report, we extend these findings by demonstrating that our severely underweight patient exhibited a markedly reduced volume of distribution (∼2–3 L) compared with reported values in normal-weight (3–4 L), overweight (4–5 L), and obese individuals (∼6–7 L).^9^ As a result, severely underweight patients may theoretically be at risk of drug overexposure when treated with the conventional 1500 mg dalbavancin dose, particularly in the context of prolonged or chronic therapy, although no plasma concentration threshold for toxicity has yet been established.

An additional key finding of this study is the demonstrated robustness and feasibility of proactive TDM of dalbavancin, based on serial Cmin and Cmax assessments.^3,4^ Through log-linear regression models, this approach reliably guided the timing of dalbavancin re-dosing, not only in individuals with obesity, as shown in prior research,^9^ but also in this severely underweight patient, maintaining Cmin concentrations consistently above the target threshold of 8 mg/L.^3–7^

In conclusion, this case report contributes valuable insight into the pharmacokinetics of dalbavancin, emphasizing that both obesity and severe underweight status can significantly influence drug distribution and dosing strategies. These findings underscore the importance of individualized, proactive TDM to optimize treatment efficacy and safety in patients at both extremes of body weight.

## Data Availability

Data are available from the corresponding author upon reasonable request.
